# Fluoroquinolones Protective against Cephalosporin Resistance in Gram-negative Nosocomial Pathogens

**DOI:** 10.3201/eid1001.020663

**Published:** 2004-01

**Authors:** Mitchell J. Schwaber, Sara E. Cosgrove, Howard S. Gold, Keith S. Kaye, Yehuda Carmeli

**Affiliations:** * Beth Israel Deaconess Medical Center and Harvard Medical School, Boston, Massachusetts, USA; † Duke University Medical Center, Durham, North Carolina, USA; ‡ Tel Aviv Sourasky Medical Center, Tel Aviv, Israel

**Keywords:** Fluoroquinolones, cephalosporins, antimicrobial drug resistance, gram-negative bacterial infections, nosocomial infections, *Enterobacter*, *Pseudomonas aeruginosa*, *Klebsiella pneumoniae*

## Abstract

In a matched case-control study, we studied the effect of prior receipt of fluoroquinolones on isolation of three third-generation cephalosporin-resistant gram-negative nosocomial pathogens. Two hundred eighty-two cases with a third-generation cephalosporin-resistant pathogen (203 with *Enterobacter* spp., 50 with *Pseudomonas* *aeruginosa,* and 29 with *Klebsiella* *pneumoniae*) were matched on length of stay to controls in a 1:2 ratio. Case-patients and controls were similar in age (mean 62 years) and sex (54% male). Variables predicting third-generation cephalosporin resistance were surgery (p = 0.005); intensive care unit stay (p < 0.001); and receipt of a β-lactam/β-lactamase inhibitor (p < 0.001), a ureidopenicillin (p = 0.002), or a third-generation cephalosporin (p < 0.001). Receipt of a fluoroquinolone was protective against isolation of a third-generation cephalosporin-resistant pathogen (p = 0.005). Interventional studies are required to determine whether replacing third-generation cephalosporins with fluoroquinolones will be effective in reducing cephalosporin resistance and the effect of such interventions on fluoroquinolone resistance.

Resistance to third-generation cephalosporins in gram-negative nosocomial pathogens is a formidable problem, associated with adverse clinical outcomes and increased hospital costs (*1*–*4*). Measures to combat the emergence and spread of resistant nosocomial pathogens have met with varying degrees of success. Although good infection control practices are the most important measure in limiting the spread of resistance, other measures are required, including changes in antimicrobial drug–prescribing patterns through formulary modification and enhanced education of prescribers (*5*).

 Kaye et al. reported a protective effect of fluoroquinolone use against the emergence of resistance to third-generation cephalosporins in nosocomial isolates of *Enterobacter* (*6*). In our study, we aimed to determine whether this protective effect is translated into an ecologic phenomenon by using individual patient-level data, i.e., whether fluoroquinolone use, in addition to lowering the likelihood of emergence of resistance in an individual patient, also results in reduced initial isolation of resistant strains in a given population. In addition, we aimed to determine whether the effect of fluoroquinolone use on *Enterobacter* spp. is applicable to other gram-negative pathogens. We conducted a matched case-control study to test the protective effect of fluoroquinolone use on the subsequent isolation of the three most common gram-negative hospital pathogens that are resistant to third-generation cephalosporins, *Enterobacter* spp., *Pseudomonas aeruginosa,* and *Klebsiella pneumoniae* (*4*).

## Methods

### Hospital Setting, Study Design, and Microbiology

During the study period, Beth Israel Deaconess Medical Center, West Campus, was a 320-bed, urban, tertiary-care teaching hospital, with 24 intensive care unit beds and approximately 12,000 admissions annually; the hospital serves a nonobstetric adult population in Boston, Massachusetts. Data were collected from administrative, laboratory, and pharmacy databases within this hospital by using relational database software (Access97, Microsoft, Redmond, WA). The microbiology database was searched to identify all cultures positive for nosocomial third-generation cephalosporin-resistant *Enterobacter* spp., *P. aeruginosa,* and *K. pneumoniae* in hospitalized patients from October 1, 1993, to June 1, 1998. To qualify for inclusion, an isolate had to grow from a culture taken no earlier than the host patient’s second hospital day. For *Enterobacter* spp. and *K. pneumoniae*, third-generation cephalosporin resistance was defined as an MIC of ceftriaxone or ceftazidime of >16 μg/mL; resistance in *P. aeruginosa* was defined as an MIC of ceftazidime of >16 μg/mL.

Patients whose clinical culture data demonstrated an isolate with the above criteria were considered case-patients. A patient could be included only once. To meet the criteria of appropriate selection of the reference group, which require that controls be derived from the same source population that gives rise to the cases (*7*), controls were selected randomly from hospitalized patients who did not have a positive culture for the studied organisms. Controls were matched to the cases in a 2:1 ratio on the basis of length of hospital stay until the positive culture was taken; thus at the time of matching, each control had been hospitalized as long as his or her index case-patient. This length of stay was characterized as the risk period.

Variables studied included patient demographics (age and sex), coexisting conditions (number of conditions, AIDS, diabetes mellitus, cardiovascular disease, hepatic disease, pulmonary disease, renal disease, and malignancy), hospital events during the risk period (surgery, intensive care unit stay), and receipt before the day of culture, for at least 24 hours, of an agent from any of the following antimicrobial drug classes: β-lactam/β-lactamase inhibitor combinations (mostly ampicillin/sulbactam and piperacillin/tazobactam), aminoglycosides (mostly gentamicin and tobramycin), first- or second-generation cephalosporins, third-generation cephalosporins (mostly ceftriaxone and ceftazidime), imipenem, ureidopenicillins (mostly piperacillin), and fluoroquinolones (mostly ciprofloxacin and ofloxacin). The route of administration of the antimicrobial agents was not considered, since the route was parenteral for all classes studied except fluoroquinolones. For fluoroquinolones, the nearly equivalent bioavailability between the oral and parenteral routes obviated the need to distinguish patients who received agents from this class orally from those who received them parenterally.

### Statistical Analysis

Statistical analyses were performed by using SAS software (SAS Institute, Inc., Cary, NC, version 8e). Matched analyses were conducted by using a conditional logistic regression model. Variables with a p value of <0.05 on univariate matched analysis were included in a multiple conditional logistic regression model. Effect modification between factors was searched for by testing appropriate interaction terms for statistical significance. Effect estimates in the regression model were reported as hazard ratios; p values of <0.05 were considered significant.

## Results

### Demographics, Coexisting Conditions, and Hospital Events

Two hundred eighty-two patients with third-generation cephalosporin-resistant nosocomial target pathogens were enrolled in the study: *Enterobacter* spp. were isolated from 203 patients, *P. aeruginosa* from 50, and *K. pneumoniae* from 29. For all but two of these case-patients, two matched controls were enrolled per case; for each of the remaining two, one control was enrolled. Thus, 562 matched controls were included. Median length of stay before enrollment in the study was 12 days. Case-patients and controls were similar in age (mean 62.4 vs. 62.1 years; p = 0.82) and sex distribution (55.3% vs. 52.7% male; p = 0.44). Characteristics of the study patients and the matched univariate comparisons for case-patients and controls are summarized in Table 1. Case-patients had a significantly higher number of coexisting conditions than controls (hazard ratio [HR] 1.22; p = 0.01); specifically, case-patients had a higher prevalence of hepatic disease (HR 1.70; p = 0.004), pulmonary disease (HR 1.52; p = 0.04), and renal disease (HR 1.71; p = 0.003). Case-patients were significantly more likely than controls to have been in an intensive care unit (HR 2.65; p < 0.001) and to have had surgery (HR 2.03; p < 0.001) during the risk period.

**Table 1:** Characteristics of study patients and univariate analysis of outcome^a^

Characteristic	Case-patients (n = 282) (%)	Controls (n = 562) (%)	HR (95% CI)^b^	p
Mean age (y)	62.4	62.1	1.00 (0.99 to 1.01)	0.82
Male	156 (55.3)	296 (52.7)	1.12 (0.84 to 1.50)	0.44
No. of coexisting conditions	0:14 (5.0)	0:47 (8.4)	1.22 (1.04 to 1.44)	0.01
	1:71 (25.2)	1:155 (27.6)		
	2:111 (39.4)	2:228 (40.6)		
	>3:86 (30.5)	>3:132 (23.5)		
AIDS	1 (0.4)	13 (2.3)	0.15 (0.02 to 1.18)	0.07
Cardiovascular disease	205 (72.7)	404 (71.9)	1.04 (0.80 to 1. 43)	0.81
Diabetes mellitus	124 (44.0)	259 (46.1)	0.92 (0.69 to 1.23)	0.57
Hepatic disease	66 (23.4)	86 (15.3)	1.70 (1.18 to 2.44)	0.004
Pulmonary disease	48 (17.0)	67 (11.9)	1.52 (1.01 to 2.28)	0.04
Renal disease	68 (24.1)	88 (15.7)	1.71 (1.20 to 2.44)	0.003
In intensive care unit during risk period	161 (57.1)	207 (36.8)	2.65 (1.91 to 3.68)	< 0.001
Malignancy	46 (16.3)	92 (16.4)	0.99 (0.67 to 1.47)	0.97
Surgery during risk period	164 (58.2)	229 (40.8)	2.03 (1.50 to 2.73)	< 0.001
Receipt of β-lactam/ β-lactamase inhibitor	111 (39.4)	125 (22.2)	2.48 (1.77 to 3.49)	< 0.001
Receipt of aminoglycoside	62 (22.0)	97 (17.3)	1.39 (0.95 to 2.04)	0.09
Receipt of 1st- or 2nd-generation cephalosporin	117 (41.5)	195 (34.7)	1.39 (1.01 to 1.92)	0.04
Receipt of third-generation cephalosporin	114 (40.4)	122 (21.7)	2.98 (2.07 to 4.27)	< 0.001
Receipt of imipenem	27 (9.6)	37 (6.6)	1.51 (0.87 to 2.62)	0.14
Receipt of ureidopenicillin	42 (14.9)	32 (5.7)	2.91 (1.77 to 4.77)	< 0.001
Receipt of fluoroquinolone	23 (8.2)	79 (14.1)	0.48 (0.28 to 0.82)	0.008

^a^Outcome refers to the isolation of third-generation cephalosporin-resistant *Enterobacter* spp., *Pseudomonas aeruginosa,* or *Klebsiella pneumoniae* from a clinical specimen. ^b^HR, hazard ratio; CI, confidence interval.

### Antimicrobial Drug Exposures

In the univariate analysis, case-patients were significantly less likely than controls to have received a fluoroquinolone (HR 0.48; p = 0.008). Case-patients were significantly more likely than controls to have received a β-lactam/β-lactamase inhibitor (HR 2.48; p < 0.001), a first- or second-generation cephalosporin (HR 1.39; p = 0.04), a third-generation cephalosporin (HR 2.98, p < 0.001), or a ureidopenicillin (HR 2.91, p < 0.001). There was also a trend toward greater use of aminoglycosides (HR 1.39; p = 0.09) and imipenem (HR 1.51; p = 0.14) in case-patients, but these associations did not achieve significance.

### Multivariable Analysis

Results of the multivariable analysis are summarized in Table 2. Neither the total number of coexisting conditions nor the frequency of any individual condition was significantly different between cases and controls. After controlling for confounding variables, however, both hospital events examined (surgery and intensive care unit exposure) remained significantly associated with the isolation of a resistant gram-negative organism (HR 1.62; p = 0.005, and HR, 2.17; p < 0.001, respectively). Three antimicrobial drug classes remained significantly associated with isolation of a resistant pathogen: β-lactam/β-lactamase inhibitor combinations (HR, 2.52; p < 0.001), ureidopenicillins (HR, 2.55; p = 0.002), and third-generation cephalosporins (HR, 2.84; p < 0.001).

**Table 2:** Multivariable analysis of outcome^a^

Characteristic	HR (95% CI)^b^	p
Surgery during risk period	1.62 (1.16 to 2.25)	0.005
In intensive care unit during risk period	2.17 (1.49 to 3.16)	<0.001
Receipt of β-lactam/β-lactamase inhibitor	2.52 (1.67 to 3.80)	<0.001
Receipt of ureidopenicillin	2.55 (1.43 to 4.53)	0.002
Receipt of 3rd-generation cephalosporin	2.84 (1.89 to 4.27)	<0.001
Receipt of fluoroquinolone	0.40 (0.21 to 0.76)	0.005

^a^Outcome refers to the isolation of third-generation cephalosporin-resistant *Enterobacter* spp., *Pseudomonas aeruginosa,* or *Klebsiella pneumoniae* from a clinical specimen. ^b^HR, hazard ratio; CI, confidence interval.

 The only factor protective against isolation of a third-generation cephalosporin-resistant gram-negative pathogen was exposure to a fluoroquinolone. After controlling for confounding, the protective effect was even more pronounced than on univariate analysis (HR, 0.4; p = 0.005). Subgroup analyses that used the same multivariable model showed a similar protective effect for fluoroquinolones against isolation of each of the three pathogens considered individually, though in the smaller two subgroups the results did not achieve significance.

 Confounding by severity of illness was controlled for in the analysis by the inclusion in the final model of intensive care unit stay and surgery before culture, as both of these hospital events, particularly the former, are markers of disease severity. None of the individual coexisting conditions analyzed, nor the total number of such conditions, differed significantly between cases and controls on univariate analysis, and thus they were not included in the final model. Moreover, forcing the term for total coexisting conditions into the multivariable model expressly to control for confounding did not change the results for any of the significant terms.

 Interaction terms between the following factors were analyzed: fluoroquinolone use and cephalosporin use, surgery and intensive care unit exposure, fluoroquinolone use and diabetes mellitus, and fluoroquinolone use and renal disease. None of these interaction terms achieved significance, and thus they were not included in the final model.

## Discussion

 Resistance to third-generation cephalosporins among gram-negative nosocomial pathogens is associated with increased mortality, length of stay, and hospital costs (*1*–*4*). Measures to reduce the extent of resistance are therefore warranted.

 This study was designed to test the hypothesis that recipients of fluoroquinolones are protected against infection and colonization with the three most common third-generation cephalosporin-resistant gram-negative nosocomial pathogens, *Enterobacter* spp., *P. aeruginosa,* and *K. pneumoniae* (*4*). We have demonstrated a protective effect of fluoroquinolone use on infection or colonization with these resistant organisms both in crude analysis and after control for confounding variables. Moreover, subgroup analysis demonstrated this protective effect for each genus individually, though small numbers of patients with cultures positive for *P. aeruginosa* and *K. pneumoniae* precluded statistical significance in these groups, due to the limited power associated with subgroup analysis. Other notable findings are that surgery, intensive care unit stay, and receipt of a β-lactam/β-lactamase inhibitor combination, a ureidopenicillin, or a third-generation cephalosporin increase the likelihood of recovery of these resistant pathogens. Although we did not match case-patients and controls based on date of admission, division of the entire study period into three time intervals showed the ratio of cases to controls to be approximately the same in each. The likelihood of spurious associations resulting from disparity between the year of hospitalization of cases and controls is therefore minimal.

 Our analysis did not differentiate between infection and colonization with the pathogens studied. Since the focus of the study was the occurrence of third-generation cephalosporin-resistant nosocomial organisms in the population we studied, this distinction was not necessary. The organisms we studied are capable of causing infection in a given patient at any point after colonization. Moreover, once they have colonized a patient, they are capable of transmission to other hospitalized patients, in whom they can cause infection. Our objective, then, was not to compare rates of active disease between hospitalized groups, but rather to use the recovery of these organisms as a marker for actual or potential disease in the populations we examined.

In addition to infection control measures, such as active surveillance, hygiene, and isolation precautions, the other important strategy in checking the emergence and spread of antimicrobial resistance is the manipulation of selective antimicrobial pressure through changes in use of antimicrobial drugs (*5*). Previous studies exploring the effect of antibiotics on third-generation cephalosporin resistance focused on replacement of cephalosporins with other β-lactam–containing agents (*8*–*11*). No interventions involving a substitution with a fluoroquinolone have been reported.

Two main categories of β-lactamases mediate resistance to third-generation cephalosporins among the common gram-negative nosocomial pathogens: chromosomal β-lactamases and plasmid-associated extended-spectrum β-lactamases (ESBLs) (*12*). Enzymes that can confer resistance to most penicillins, cephalosporins, and monobactams, ESBLs belong to Bush-Jacoby-Medeiros functional group 2, whose enzymes are generally inhibited in vitro by β-lactamase inhibitors. By contrast, the chromosomal β-lactamases present in *Enterobacter* and *Pseudomonas* (which constitute 90% of the resistant isolates in our study) belong to group 1, whose enzymes are not inhibited by β-lactamase inhibitors (*13*).

 Earlier studies described interventions carried out when plasmid-associated ESBLs were the main mechanism of resistance, so it is not surprising that replacing cephalosporins with a β-lactam/β-lactamase inhibitor combination, as was done in some of these studies (*8*,*9*,*11*), resulted in reduced rates of cephalosporin resistance. Our study, by contrast, found both ureidopenicillins and β-lactam/β-lactamase inhibitor combinations to be risk factors for the isolation of gram-negative organisms resistant to third-generation cephalosporins. We believe that this discrepancy relates to the fact that the predominant cause of resistance in our hospital during the study period was group 1 chromosomal β-lactamases (against which β-lactamase inhibitors are not active) and that plasmid-mediated ESBLs played only a minimal role (*14*).

 Our findings expand on the observations of Kaye et al. regarding the protective effect of fluoroquinolones on the emergence of third-generation cephalosporin-resistant *Enterobacter* spp (*6*). They diverge, however, regarding risk factors. Although Kaye et al. found third-generation cephalosporin exposure to be an independent risk factor for emergence of resistance; no other antimicrobial exposure or hospital event was independently associated with this finding. We propose that the suggested discrepancy between these results and our findings that certain hospital events and antimicrobial classes confer enhanced risk for initial isolation of resistant organisms can be attributed to the difference in the design of the two studies.

Kaye et al., in examining emergence of resistance, identified clinical isolates for which β-lactamase production was induced or derepressed mutants were selected. Our study design, by contrast, detected those patients colonized or infected by an organism with preexisting third-generation cephalosporin resistance, a phenomenon made more likely by certain hospital events or antimicrobial drug exposures. Whereas Kaye’s case-patients began with susceptible isolates that developed resistance after a specific exposure, our case-patients were enrolled with already resistant strains. Thus, while a particular hospital event or antimicrobial drug exposure may not induce β-lactamase production or select derepressed mutants, it may well confer enhanced susceptibility to the acquisition of a strain in which resistance mechanisms are already expressed.

Although we do not include molecular typing or epidemiologic data regarding patterns of antimicrobial drug use and colonization with resistant organisms, earlier studies conducted during our study period at the same institution have answered many of these questions (*14*,*15*). These studies showed that colonization with ceftazidime-resistant gram-negative bacilli in intensive care units during a nonoutbreak period was common, was probably acquired before admission to the unit, involved diverse strains, and was associated with prior exposure to a variety of β-lactam antimicrobial drugs.

When one generalizes from the interventional studies performed to date, replacing third-generation cephalosporins with other agents is difficult because they are prone to several possible biases: 1) they are before/after studies and are therefore prone to time effect bias; 2) they describe a group-level analysis and are therefore prone to ecologic bias (*16*); 3) the formulary intervention is usually coupled with improved infection control measures, causing difficulty in determining which measure is responsible for the noted effect; and 4) these studies are more likely to be reported and published when a positive effect is noted, i.e., publication bias.

Our data as well as those of Kaye et al. suggest that fluoroquinolones could be substituted for certain types of β-lactam antimicrobial drugs to prevent the emergence and lower the rates of isolation of the most common third-generation cephalosporin-resistant gram-negative nosocomial pathogens. The potential advantages of adding fluoroquinolones to the armamentarium of agents that can be used to combat third-generation cephalosporin resistance are several: they can be administered orally; they are relatively nontoxic and inexpensive; and they may allow the replacement of earlier generation cephalosporins, receipt of which has previously been identified as a risk factor for isolation of third-generation cephalosporin-resistant gram-negative organisms (*15*).

A limitation of retrospective analyses is the inability to prove what appear to be causal relationships. Statistical associations are interpreted as risk factors, and inverse associations as protective effects. Proof that fluoroquinolones are in fact protective against the isolation of nosocomial third-generation cephalosporin-resistant gram-negative pathogens, as suggested by the inverse association demonstrated here, will require animal models or prospective interventional studies. Such studies will also be required to determine whether reduced third-generation cephalosporin resistance will come at the cost of increased levels of fluoroquinolone resistance, a phenomenon to which Burke has referred as “squeezing the balloon” (*17*). Fluoroquinolone resistance, not addressed in our study, occurs primarily by means of chromosomal mutation (*18*), and resistant mutants could potentially be selected for by increased use of this class of antimicrobial agent. Our data, then, provide the impetus for further studies, including a prospective interventional trial to explore the overall protective efficacy of fluoroquinolones against multiresistant gram-negative pathogens.
